# Characterization of the Intestinal Absorption of Seven Flavonoids from the Flowers of *Trollius chinensis* Using the Caco-2 Cell Monolayer Model

**DOI:** 10.1371/journal.pone.0119263

**Published:** 2015-03-19

**Authors:** Lijia Liu, Lina Guo, Can Zhao, Xiuwen Wu, Rufeng Wang, Chen Liu

**Affiliations:** School of Chinese Materia Medica, Beijing University of Chinese Medicine, Beijing, China; National Institute of Agronomic Research, FRANCE

## Abstract

The human Caco-2 cell monolayer model was used to investigate the absorption property, mechanism, and structure-property relationship of seven representative flavonoids, namely, orientin, vitexin, 2”-*O*-β-L-galactopyranosylorientin, 2”-*O*-β-L-galactopyranosylvitexin, isoswertisin, isoswertiajaponin, and 2”-*O*-(2”‘-methylbutanoyl)isoswertisin from the flowers of *Trollius chinensis*. The results showed that these flavonoids were hardly transported through the Caco-2 cell monolayer. The compounds with 7-OCH_3_ including isoswertisin, isoswertiajaponin and 2”-*O*-(2”‘-methylbutanoyl)isoswertisin were absorbed in a passive diffusion manner, and their absorbability was increased in the same order as their polarity. The absorption of the remaining compounds with 7-OH including orientin, vitexin, 2”-*O*-β-L-galactopyranosylorientin, and 2”-*O*-β-L-galactopyranosylvitexin involved transporter mediated efflux in addition to passive diffusion. Among the four compounds with 7-OH, those with a free hydroxyl group at C-2” such as orientin and vitexin were the substrates of P-glycoprotein (P-gp) and that with a free hydroxyl group at C-2’ such as 2”-*O*-β-L-galactopyranosylorientin was the substrate of multidrug resistance protein 2 (MRP2). The results of this study also implied that the absorbability of the flavonoids should be taken into account when estimating the effective components of *T*. *chinensis*.

## Introduction

**Fig 1:**
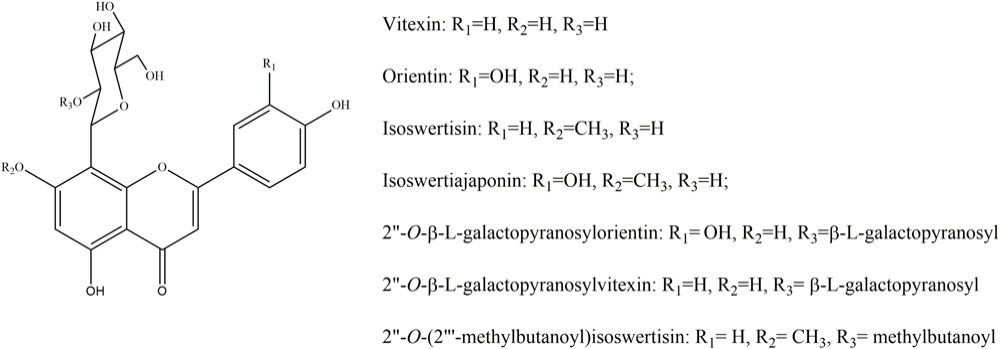
The structures of the seven flavonoids.

The flowers of *Trollius chinensis*, which mainly contain flavonoids, phenolic acids and alkaloids [1–2], have been used by Chinese people for the treatment of respiratory infections, pharyngitis, tonsillitis, and bronchitis since ancient time [3]. Flavonoids are the most abundant compounds in these flowers and account for approximately 16 percent of the total dried weight of the whole flower [4]. The overwhelming majority of the flavonoids found in the flowers are flavone *C*-glycosides which are formed by the sugar moieties and flavone aglycones at C-8 through C-C glycosidic bond. These *C*-glycosides can be categorized into two groups on the basis of their structure. One group is composed of orientin, vitexin and their derivatives such as 2”-*O*-β-L-galactopyranosylorientin and 2”-*O*-β-L-galactopyranosylvitexin, which are characterized by a hydroxy at C-7 of the flavone skeleton [5–11]. Another is composed of isoswertiajaponin, isoswertisin and their derivatives such as 2”-*O*-(2”‘-methylbutanoyl)isoswertisin, which are characterized by a methoxy at C-7 of the flavone skeleton [10–12]. Modern Pharmacological studies have proven that flavonoids including above compounds from the flowers of *T*. *chinensis* possess antiviral, antibacterial, and anti-inflammatory activities *in vitro*. The major flavonoids orientin and vitexin exhibited potent or moderate antiviral activities against parainfluenza virus type 3 (Para 3) with the IC_50_ values of 11.7 and 20.8 μg/ml [13], respectively, and 2”-*O*-(2”‘-methylbutanoyl)isoswertisin had an effect against influenza virus A [12]. Orientin showed potent inhibitory effect against both *Staphylococcus aureus* and *S*. *epidermidis* with the MIC values of 25 mg/L, and vitexin demonstrated potent to moderate effect against those two bacteria with the MIC values of 100 mg/L toward *S*. *aureus* and 25 mg/L toward *S*. *epidermidis*, respectively [14–17]. 2”-*O*-(2”‘-methylbutanoyl)isoswertisin exhibited anti-inflammatory effect with an inhibitory rate of 35.5% on murine ear edema model [18], and orientin and vitexin were also proven by our research group to possess anti-inflammatory effects against murine ear edema [19]. Most investigators believe that flavonoids, due to their abundance and bioactivities *in vitro*, are the principal effective substances of the flowers of *T*. *chinensis*. However, flavone *C*-glycosides are usually considered of low bioavailability. As is well known, Chinese herbal drugs are usually used through oral administration. This renders intestinal absorption very crucial to the evaluation of the bioactivity of these flavone *C*-glycosides and their contributions to the efficacy of these flowers. For this reason, we selected the seven flavonoids mentioned above (Fig. 1) to investigate their absorption properties and mechanisms, as well as the structure-absorbability relationship using the well-recognized human Caco-2 cell monolayer model, so as to predict their bioavailability and facilitate the correct understanding of their contribution to the efficacy of these flowers.

## Materials and Methods

### Reagents and instruments

Orientin (2-(3,4-dihydroxyphenyl)-5,7-dihydroxy-8-((2*S*,3*R*,4*R*,5*S*,6*R*)-3,4,5-trihydroxy-6-(hydroxymethyl)tetrahydro-2*H*-pyran-2-yl)-4*H*-chromen-4-one), vitexin (5,7-dihydroxy-2-(4-hydroxyphenyl)-8-((2*S*,3*R*,4*R*,5*S*,6*R*)-3,4,5-trihydroxy-6-(hydroxymethyl)tetrahydro-2*H*-pyran-2-yl)-4*H*-chromen-4-one), 2”-*O*-β-L-galactopyranosylvitexin (8-((2*S*,3*R*,4*S*,5*S*,6*R*)-4,5-dihydroxy-6-(hydroxymethyl)-3-(((2*S*,3*R*,4*S*,5*R*,6*R*)-3,4,5-trihydroxy-6-(hydroxymethyl)tetrahydro-2*H*-pyran-2-yl)oxy)tetrahydro-2*H*-pyran-2-yl)-5,7-dihydroxy-2-(4-hydroxyphenyl)-4*H*-chromen-4-one), isoswertisin (5-hydroxy-2-(4-hydroxyphenyl)-7-methoxy-8-((2*S*,3*R*,4*R*,5*S*,6*R*)-3,4,5-trihydroxy-6-(hydroxymethyl)tetrahydro-2*H*-pyran-2-yl)-4*H*-chromen-4-one), isoswertiajaponin (2-(3,4-dihydroxyphenyl)-5-hydroxy-7-methoxy-8-((2*S*,3*R*,4*R*,5*S*,6*R*)-3,4,5-trihydroxy-6-(hydroxymethyl)tetrahydro-2*H*-pyran-2-yl)-4*H*-chromen-4-one), and 2”-*O*-(2”‘-methylbutanoyl)isoswertisin ((2*S*,3*R*,4*S*,5*S*,6*R*)-4,5-dihydroxy-2-(5-hydroxy-2-(4-hydroxyphenyl)-7-methoxy-4-oxo-4*H*-chromen-8-yl)-6-(hydroxymethyl)tetrahydro-2*H*-pyran-3-yl 2-methylbutanoate) were isolated from the flowers of *T*. *chinensis* by our research group [10, 12], 2”-*O*-β-L-galactopyranosylorientin (8-((2*S*,3*R*,4*S*,5*S*,6*R*)-4,5-dihydroxy-6-(hydroxymethyl)-3-(((2*S*,3*R*,4*S*,5*R*,6*R*)-3,4,5-trihydroxy-6-(hydroxymethyl)tetrahydro-2*H*-pyran-2-yl)oxy)tetrahydro-2*H*-pyran-2-yl)-2-(3,4-dihydroxyphenyl)-5,7-dihydroxy-4*H*-chromen-4-one) was purchased from Chengdu Puruifa Technology Development Company (Chengdu, China), and verapamil hydrochloride and probenecid were obtained from National Institutes for Food and Drug Control (Beijing, China). The purities of all of the above substances were over 98% based on their label or determined by our High Performance Liquid Chromatography (HPLC) assay. Dimethyl sulfoxide (DMSO), propranolol and atenolol with the purity of minimum 98% were products of Sigma Chemical Co. (Deisenhofen, Germany). Dulbecco’s Modified Eagle’s Medium (DMEM), fetal bovine serum (FBS) and trypsin were supplied by Gibco (Grand Island, CA, USA). Nonessential amino acids (NEAA), penicillin and streptomycin were purchased from Corning (Cambridge, MA, USA). 3-(4,5-dimethylthiazolzyl)-2,5-diphenyl tetrazolium bromide (MTT) was produced by Amresco (Radnor, PA, USA). Paclitaxel was the product of Beijing SL Pharmaceutical Co., Ltd (Beijing, China). Acetonitrile was of HPLC grade (Fisher Co. Pittsburgh, PA, USA), and acetic acid was of analytical grade (Beijing Chemical Factory, Beijing, China).

Transwell plates (insert diameter 12 mm, pore size 3.0 μm, membrane growth area 1.12 cm^2^) and 96-wells plates were obtained from Corning Costar (Cambridge, MA, USA). Millicell-ERS system was the product of Millipore Corp. (Bedford, OH, USA). HPLC analysis was performed on a Waters 1500 series equipped with a 1525 Binary HPLC pump, an on-line degasser, and a 2489 UV/Visible detector using Kromasil C_18_ ODS column (250 mm×4.60 mm i.d., 5 μm particle size) with a guard column. The signal was acquired and processed into chromatogram using a Waters Breeze 2 software. JB-CJ-1500 super clean bench (Beijing Great Wall Air Purification Co. Ltd., Beijing, China), MCO-18AIC CO_2_ incubator (SANYO Inc., Osaka, Japan), HYG-B thermostatic oscillator (Beijing Jiayuan Xingye Technology Ltd., Beijing, China), Epoch microplate reader (BioTek Instruments, Inc., Winooski, VT, USA), and TGL-16C high speed tabletop centrifuge (Shanghai Anting Scientific Instruments Plant, Shanghai, China) were also used in this study.

### Cell culture

The human colon adenocarcinoma cell line Caco-2 (No. 3111C0001CCC000100, passage 32) was obtained from the Cell Resource Center, Peking Union Medical College (the headquarters of National Infrastructure of Cell Line Resource, NSTI) on May 3rd, 2013. The cell line was checked free of mycoplasma contamination by PCR and culture, its species origin was confirmed with PCR, and its identity was authenticated with STR profiling (FBI, CODIS). All results of the above tests can be viewed on the website (http://cellresource.cn). The cells were cultured in 25 cm^2^ flasks in DMEM medium supplemented with 10% (v/v) FBS, 1% (v/v) NEAA, and 1% (v/v) penicillin-streptomycin, in an atmosphere of 5% CO_2_ at 37°C and constant moisture. The culture medium was replaced every 2 to 3 days, and the cells were split 1:2 or 1:3 as 80% confluence was obtained.

### MTT assay

Caco-2 cells at logarithmic growth phase were seeded into 96-well plates and incubated at 37°C in an atmosphere of 5% CO_2_ and constant moisture in the incubator. After the cells reached confluence, they were cultured in quadruplicate with 100 μL of HBSS solution (negative control) and compound-containing HBSS solution (seven flavonoids or paclitaxel (positive control) at the concentrations of 25, 50, and 100 μmol/L, respectively) for 4 h. Then, HBSS solution was replaced by 100 μL of MTT solution (0.5 mg/mL), and the supernatant was discarded after 4 h of incubation at 37°C. The cells were shaken with 150 μL of DMSO for 10 min, and the absorbance was read on the microplate reader at 570 nm. The survival rate $\%=\bar{\text{A}}/\bar{{\text{A}}_{0}}\times 100$, wherein $\bar{\text{A}}$ was the average absorbance value of treatment or positive control group, and $\bar{{\text{A}}_{0}}$ was the average absorbance value of negative control group.

### Cell differentiation

For cells differentiation to form a confluent monolayer, Caco-2 cells were seeded into the inserts of the 12-well Transwell plates at the density of 1.0×10^5^ cells/cm^2^. The culture medium was replaced on every other day in the first week, and then at daily intervals for the apical (AP) side and 2 days intervals for the basal (BL) side until the transport experiment was performed 21 days after seeding. The AP and BL sides contained 0.5 and 1.5 mL of culture medium, respectively. The integrity and viability of the cell monolayers were evaluated by measuring transepithelial electrical resistance (TEER) values between AP and BL sides with Millicell-ERS system and transport experiment using the standard compounds, i.e., propranolol and atenolol which were well-recognized control substances for high and poor transcellular transport markers, respectively. The cell inserts were considered as qualified only if the resistance reached above 600 Ω cm^2^.

### Transport experiment

On day 21, the transport experiment was initiated by removal of the culture medium from AP and BL sides. The Caco-2 monolayers were rinsed twice with pre-warmed Hanks balanced salt solution (HBSS) and were incubated with the same solution at 37°C for 30 min. The seven individual test compounds and an equimolar mixture of them were pre-dissolved in DMSO, and then diluted to 25, 50, and 100 μmol/L with an appropriate volume of HBSS to allow the final concentration of DMSO to be less than 1%. The test compounds were added to the AP (0.5 mL) or BL side (1.5 mL), while the receiving chamber contained the corresponding volume of HBSS. Incubation was performed at 37°C for 180 min, with shaking at 50 rpm. At 30, 60, 90, 120, 150, and 180 min, each 0.2 mL of the solution from BL or AP side was collected, and replaced with an equal volume of HBSS. The samples were frozen immediately and stored below-20°C before analysis.

### Transporter inhibition experiment

On day 21, transport study was initiated by carefully removing the culture medium from both AP and BL sides. The Caco-2 monolayers were rinsed twice with pre-warmed HBSS and were incubated with the same solution at 37°C for 30 min. Verapamil hydrochloride and probenecid were dissolved in DMSO, and then added into the 50 μmol/L orientin, vitexin, 2”-*O*-β-L-galactopyranosylorientin, and 2”-*O*-β-L-galactopyranosylvitexin before use and their final concentrations were 100 and 600 μmol/L, respectively. The final concentration of DMSO in these solutions was less than 1%. The 50 μmol/L solutions of the test compounds with or without verapamil hydrochloride or probenecid were added to the AP sides (0.5 mL), and BL sides were added with 1.5 mL of HBSS. Incubation was performed at 37°C for 180 min, with shaking at 50 rpm. At 30, 60, 90, 120, 150, and 180 min, each 0.2 mL of the solution from BL side was collected, and replaced with an equal volume of HBSS. The samples were frozen immediately and stored below-20°C before analysis.

### HPLC analysis

#### Chromatographic condition

**Fig 2:**
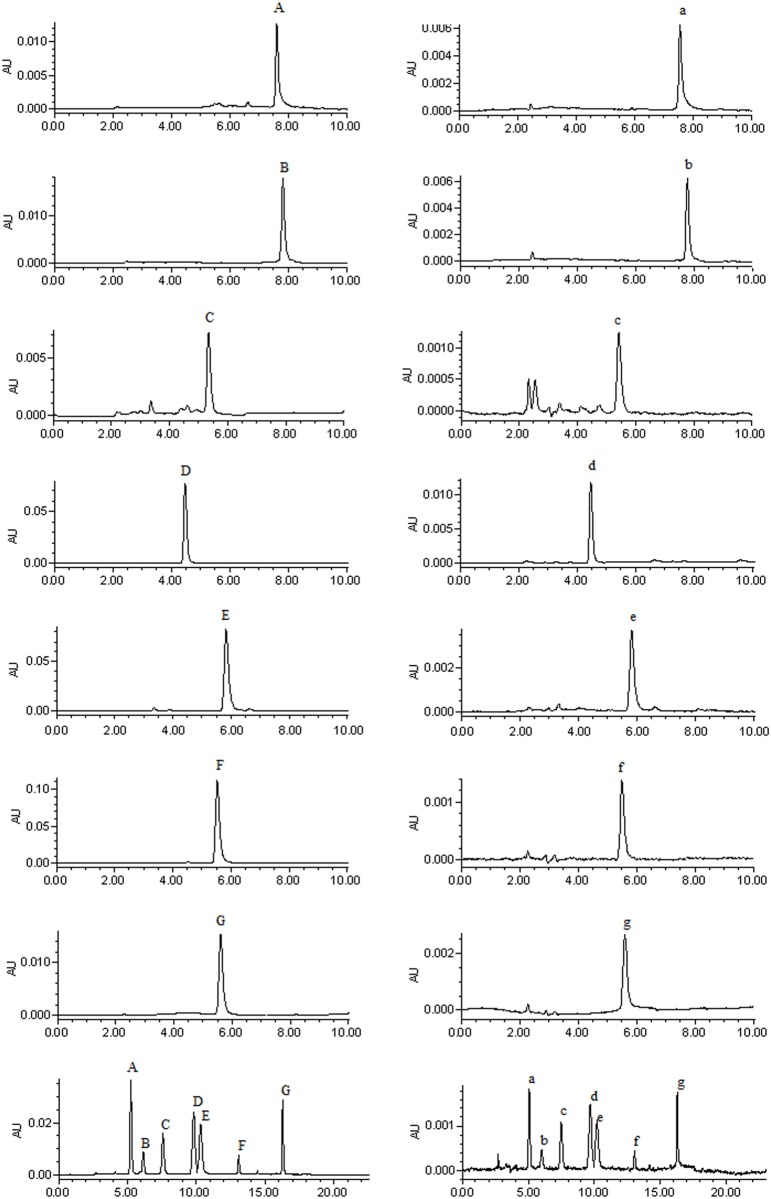
HPLC chromatograms of the seven flavonoids and their equimolar mixture. Notes: A, B, C, D, E, F, and G were chromatograms of the reference solutions of 2”-*O*-β-L-galactopyranosylorientin, 2”-*O*-β-L-galactopyranosylvitexin, orientin, vitexin, isoswertiajaponin, isoswertisin, and 2”-*O*-(2”‘-methylbutanoyl)isoswertisin; a, b, c, d, e, f, and g were chromatograms of the sample solutions of the corresponding compounds across the Caco-2 cell monolayer.

All samples were filtered through a 0.45 μm filter, and each 20 μL was used for HPLC analysis. The mobile phase used for all analytes consisted of acetonitrile (A) and 1% acetic acid (B) in different ratios, and the flow rate was constant at 1 mL/min. The chromatographic processes for orientin, isoswertiajaponin, isoswertisin, and 2”-*O*-(2”‘-methylbutanoyl)isoswertisin were performed with isocratic elution at the mobile phase ratios (A:B, v/v) of 20:80, 22:78, 25:75, and 25:75, and detecting wavelengths of 345, 350, 346, and 336 nm, respectively. The column temperature for all of these four compounds was selected at 35℃. The processes for 2”-*O*-β-L-galactopyranosylorientin and 2”-*O*-β-L-galactopyranosylvitexin were conducted with gradient elution program including 5%-30% A at 0–6 min, and 30%-5% A at 6–9 min using the column temperature of 30℃ and detecting wavelengths of 348 nm and 335 nm, respectively. For vitexin, gradient elution program including 20%-65% A at 0–7 min, and 65%-20% A at 7–9 min was employed along with the column temperature of 35℃ and the detecting wavelength of 330 nm. For the equimolar mixture of the seven flavonoids, the gradient elution program (20% A at 0–8 min, 20%-58% A at 8–14 min, 58% A at 14–17 min, and 58%-20% at 17–20 min) was conducted with the column temperature of 35℃ and the detecting wavelength of 365nm. The chromatograms for these seven flavonoids and the mixture are shown in Fig. 2.

#### Linearity, precision and stability

The calibration equations for these seven flavonoids were obtained by plotting peak area (Y, mAU*min) *vs* amount (X, in μmol). The results are as follows: for orientin, Y = 2.95×10^8^X+89 (r = 0.9998), with a good linearity over the range from 5.0×10^-7^ to 2.5×10^-4^ μmol; for vitexin, Y = 3.25×10^8^X-205 (r = 0.9998),with a good linearity over the range from 5×10^-7^ to 2.5×10^-4^ μmol; for 2”-*O*-β-L-galactopyranosylorientin, Y = 2.95×10^8^X+89 (r = 0.9998), with a good linearity over the range from 1.0×10^-6^ to 2.0×10^-4^ μmol; for 2”-*O*-β-L-galactopyranosylvitexin, Y = 4.65×10^8^X+11 (r = 0.9999), with a good linearity over the range from 5.0×10^-7^ to 5.0×10^-5^ μmol; for isoswertisin, Y = 4.51×10^8^X+97 (r = 0.9999), with a good linearity over the range from 1.0×10^-6^ to 5×10^-5^ μmol; for isoswertiajaponin, Y = 4.46×10^8^X-768 (r = 0.9997), with a good linearity over the range from 1.25×10^-6^ to 1.0×10^-4^ μmol; for 2”-*O*-(2”‘-methylbutanoyl)isoswertisin, Y = 5.23×10^8^X-619 (r = 0.9996) with a good linearity over the range from 5×10^-7^ to 1.25×10^-4^ μmol. The tailing factors for the individual peaks of orientin, vitexin, 2”-*O*-β-L-galactopyranosylorientin, 2”-*O*-β-L-galactopyranosylvitexin, isoswertinsin, isoswertiajaponin and 2”-*O*-(2”‘-methylbutanoyl)isoswertisin were 0.95, 1.07, 1.13, 1.08, 1.08, 1.04, and 0.99, respectively, and their theoretical plate numbers were 3402, 7403, 10275, 10993, 4380, 3687, and 3949, respectively.

The intra-day precision was determined by investigating the analytes in sextuplicates during a single day, and the inter-day precision was determined by conducting the intra-day experiment on two consecutive days. The RSD% values of intra-day precision for orientin, vitexin, 2”-*O*-β-L-galactopyranosylorientin, 2”-*O*-β-L-galactopyranosylvitexin, isoswertisin, isoswertiajaponin, and 2”-*O*-(2”‘-methylbutanoyl)isoswertisin were 1.43, 0.57, 0.55, 1.65, 0.36, 1.76, and 1.61, respectively, while the values of inter-day precision for these same compounds were 2.17, 1.93, 2.93, 1.68, 4.98, 2.18, and 2.83, respectively. All data demonstrated that the methods and instrument were in good condition. Each one sample solution of the seven flavonoids was kept at room temperature, and then their stabilities were determined by injecting them into apparatus at 0, 1, 2, 4, 6, 12, and 24 h. The RSD% values for 24-hour stability of the seven compounds were 2.35, 2.42, 2.86, 2.65, 2.82, 2.95, and 3.89, respectively, which showed that the samples were stable within 24 h.

### Apparent permeability coefficient and transport rate determination

The apparent permeability coefficients (P_app_) were determined in this study by HPLC quantification of the compounds in the receiver chamber after transport across the Caco-2 monolayer. The calculation was carried out in accordance with the formula P_app_ = (dQ/dt)×(1/A)×(1/C_0_), wherein dQ/dt was the rate of appearance of the test compound in the receiver compartment (μmol/s); C_0_ was the initial concentration of test compound in the donor compartment (μmol/mL); and A was the surface area of the Caco-2 monolayer (cm^2^). The percentage transported (%) was calculated using the formula % transported = Q_B_/(C_S_×V_D_)×100, wherein Q_B_ was the quantity of compound in the receiver compartment (μmol); C_S_ was the concentration of test compound in the donor compartment (μmol/mL); V_D_ was the liquid volume of the donor compartment (mL). The transport rate (μmol/h/cm^2^) was calculated using the formula transport rate = Q_B_/(t×A), wherein Q_B_ was the quantity of compound in the receiver compartment (μmol); t was the incubation time (h); A was the surface area of the Caco-2 monolayer (cm^2^).

### Statistical analysis and logP calculation

The results were presented as mean ± SD, of which the mean was the average of at least three replicates and SD was the standard deviation. The data were analyzed by either *t* test, nonparametric test or analysis of variance (ANOVA) using SPSS 16.0. The level of significance was set at *p* < 0.05. The logarithm of octanol-water partition coefficient (logP) was calculated with ChemOffice 2004 (Cambridge Soft Corporation, Cambridge, MA, USA).

## Results

### Validation of the model

After seeding, the TEER values of the monolayers developed in this study increased steadily over time, and were above 600 Ω cm^2^ on day 21. The P_app_ values of propranolol and atenolol tested with the monolayers were (1.44±0.12)×10^-5^ and (4.63±0.33)×10^-7^ cm/s, respectively, which were closely consistent with the acceptable values reported in the literature [20–23]. Thus, the Caco-2 cell monolayer model established herein was validated for the assessment of the intestinal absorption potential of the compounds of interest.

### Cell viability

The survival rates of Caco-2 cells treated with paclitaxel, which was used as a positive control for cytotoxicity, and the seven flavonoids at the concentrations of 25, 50, and 100 μmol/L for 4 h are exhibited in Table 1. As we can see, the survival rates of paclitaxel were 67.3%, 45.5%, and 37.4%, respectively, which indicated that MTT method is practicable. The survival rates for all of the seven flavonoids were over 95%, indicating that none of these compounds was toxic to Caco-2 cells at 0–100 μmol/L for 4 h. So, 25, 50, and 100 μmol/L were chosen as the test concentrations.

**Table 1:** Survival rates of Caco-2 cells treated with the seven flavonoids and paclitaxel.

Compounds	Survival rates (%)		
	25 μmol/L	50 μmol/L	100 μmol/L
Paclitaxel	67.3±8.2	45.5±6.7	37.4±3.5
Orientin	100.4±1.3	114.3±7.5	111.5±2.4
Vitexin	104.5±10.5	101.3±2.3	98.2±8.9
2”-*O*-β-L-galactopyranosylorientin	111.0±9.1	104.7±2.7	102.7±7.7
2”-*O*-β-L-galactopyranosylvitexin	110.8±12.8	117.4±6.2	105.6±5.2
Isoswertisin	110.8±9.9	115.3±5.5	116.0±8.6
Isoswertiajaponin	116.7±8.9	124.6±5.4	109.1±12.6
2”-*O*-(2”‘-methylbutanoyl)isoswertisin	108.8±10.3	127.6±4.9	128.9±12.5

### Trans-monolayer transport

The absorbability of the seven flavonoids was evaluated under the conditions of this experiment using the validated Caco-2 cell monolayer model. The transport was monitored at the concentration of 50 μmol/L for a period of 90 min. The bidirectional P_app_ values of the seven individual compounds and their equimolar mixture have been summarized in Tables 2 and 3, respectively. No matter whether the transport experiments of the seven flavonoids were conducted individually or not, the P_app_ values of orientin, vitexin, 2”-*O*-β-L-galactopyranosylorientin, 2”-*O*-β-L-galactopyranosylvitexin, isoswertisin, and isoswertiajaponin were at the level of 10^-7^, while that of 2”-*O*-(2”‘-methylbutanoyl)isoswertisin was at the level of 10^-6^.

**Table 2:** The bidirectional P_app_ values of the seven individual flavonoids and their LogP.

Compounds	P_app AP→BL_	P_app BL→AP_	P _ratio_	LogP
	(×10^-6^ cm/s)	(×10^-6^ cm/s)	P_app AP→BL_/P_app BL→AP_	
Orientin	0.75±0.05^b^, ^cc^, ^dd^	1.14±0.08^b^, ^cc^, ^dd^	0.66	-1.11
Vitexin	2.26±0.37^a^	3.38±0.54^a^, ^c^	0.67	-0.72
2”-*O*-β-L-galactopyranosylorientin	1.84±0.01^aa^	4.58±0.18^aa^, ^b^, ^dd^	0.40	-2.85
2”-*O*-β-L-galactopyranosylvitexin	1.85±0.02^aa^	4.68±0.02^aa^, ^cc^	0.39	-2.46
Isoswertisin	1.07±0.08^△△^	0.94±0.18^△△^	1.14	-0.46
Isoswertiajaponin	0.98±0.03^△△^	1.19±0.07^△△^	0.83	-0.85
2”-*O*-(2”‘-methylbutanoyl)isoswertisin	4.50±0.03**, ^##^	4.80±0.53**, ^##^	0.94	1.17

Notes: The P_app_ values were determined for a period of 90 min at the concentration of 50 μmol/L. The four compounds of which the absorption involving efflux were compared with each other:

^a^ *P*<0.05,

^aa^ *P*<0.01, compared with orientin;

^b^ *P*<0.05, compared with vitexin;

^c^ *P<0*.*05*,

^cc^ *P*<0.01, compared with 2”-*O*-β-L-galactopyranosylorientin;

^dd^ *P*<0.01, compared with 2”-*O*-β-L-galactopyranosylvitexin. And the three compounds of which the absorption mainly involving passive diffusion were compared with each other:

** *P*<0.01, compared with isoswertisin,

^##^ *P*<0.01 compared with isoswertiajaponin;

^△△^ *P*<0.01, compared with 2”-*O*-(2”‘-methylbutanoyl)isoswertisin.

**Table 3:** The bidirectional P_app_ values of the seven flavonoids in the equimolar mixture.

Compounds	P_app AP→BL_	P_app BL→AP_	P _ratio_
	(×10^-6^ cm/s)	(×10^-6^ cm/s)	P_app AP→BL_/P_app BL→AP_
Orientin	1.14±0.27^dd^	0.88±0.31	1.29
Vitexin	1.60±0.18	1.03±0.28	1.55
2”-*O*-β-L-galactopyranosylorientin	1.31±0.38^d^	0.69±0.26	1.90
2”-*O*-β-L-galactopyranosylvitexin	1.92±0.42^aa^, ^c^	0.81±0.27	2.38
Isoswertisin	2.69±0.37^△△^	1.59±0.20^##^, ^△△^	1.69
Isoswertiajaponin	1.44±0.47^△△^	0.54±0.31**, ^△△^	2.66
2”-*O*-(2”‘-methylbutanoyl)isoswertisin	3.49±0.47**, ^##^	13.7±1.14**, ^##^	0.25

Notes: The P_app_ values were determined for a period of 90 min at the concentration of 50 μmol/L. The four compounds of which the absorption involving efflux were compared with each other:

^aa^ *P*<0.01, compared with orientin;

^c^ *P*<0.05, compared with 2”-*O*-β-L-galactopyranosylorientin;

^d^ *P*<0.05,

^dd^ *P*<0.01, compared with 2”-*O*-β-L-galactopyranosylvitexin. And the three compounds of which the absorption mainly involving passive diffusion were compared with each other:

** *P*<0.01, compared with isoswertisin,

^##^ *P*<0.01 compared with isoswertiajaponin;

^△△^ *P*<0.01, compared with 2”-*O*-(2”‘-methylbutanoyl)isoswertisin.

**Fig 3:**
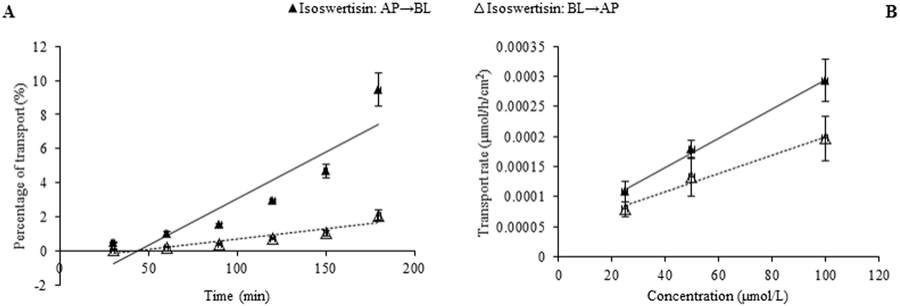
Transport of isoswertisin across Caco-2 cell monolayer. Notes: (A) The percent transport of isoswertisin as a function of time at 50 μmol/L; (B) The transport rate of isoswertisin as a function of concentration at 90 min.

**Fig 4:**
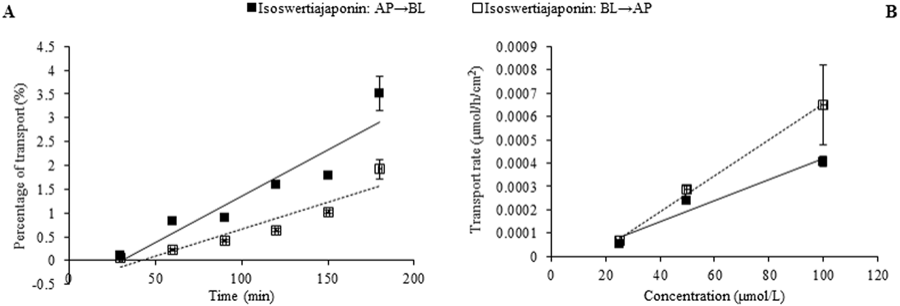
Transport of isoswertiajaponin across Caco-2 cell monolayer. Notes: (A) The percent transport of isoswertiajaponin as a function of time at 50 μmol/L; (B) The transport rate of isoswertiajaponin as a function of concentration at 90 min.

**Fig 5:**
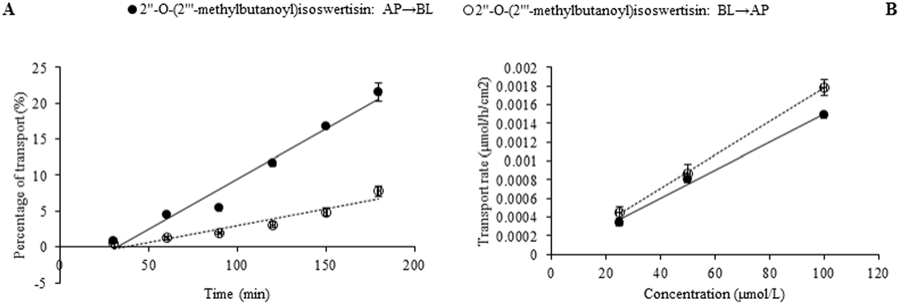
Transport of 2”-*O*-(2”‘-methylbutanoyl)isoswertisin across Caco-2 cell monolayer. Notes: (A) The percent transport of 2”-*O*-(2”‘-methylbutanoyl)isoswertisin as a function of time at 50 μmol/L; (B) The transport rate of 2”-*O*-(2”‘-methylbutanoyl)isoswertisin as a function of concentration at 90 min.

**Fig 6:**
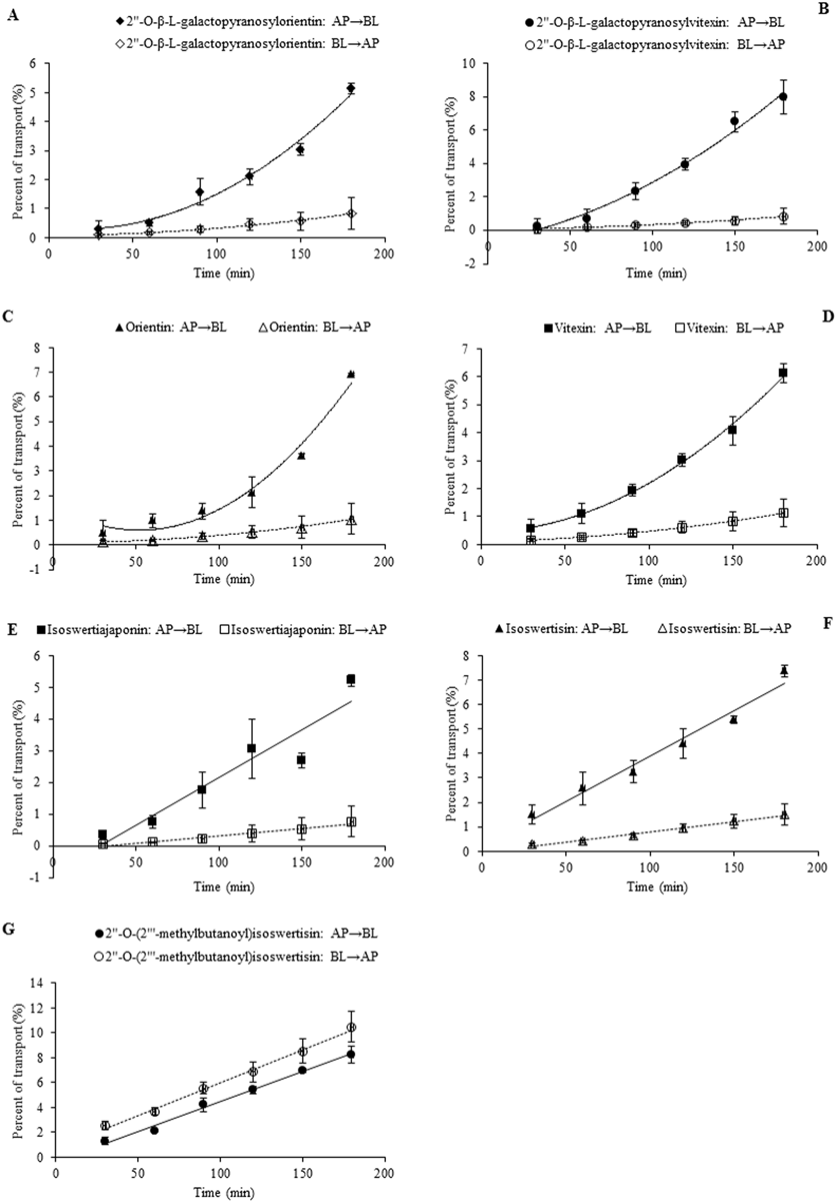
The percent transport of the seven flavonoids in the mixture as a function of time at 50 μmol/L. Notes: (A) 2”-*O*-β-L-galactopyranosylorientin; (B) 2”-*O*-β-L-galactopyranosylvitexin; (C) Orientin; (D) Vitexin; (E) Isoswertiajaponin; (F) Isoswertisin; (G) 2”-*O*-(2”‘-methylbutanoyl)isoswertisin.

**Fig 7:**
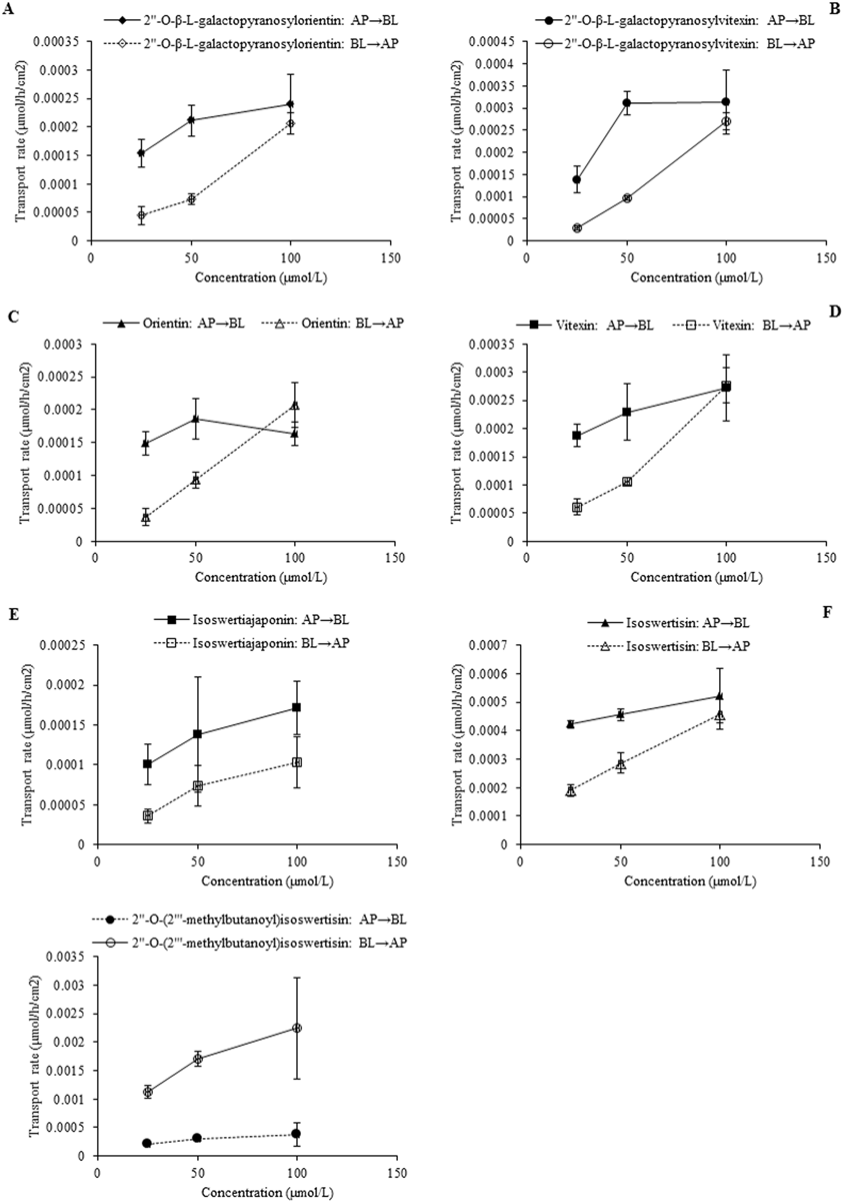
The transport rate of the seven flavonoids in the mixture as a function of concentration at 90 min. Notes: (A) 2”-*O*-β-L-galactopyranosylorientin; (B) 2”-*O*-β-L-galactopyranosylvitexin; (C) Orientin; (D) Vitexin; (E) Isoswertiajaponin; (F) Isoswertisin; (G) 2”-*O*-(2”‘-methylbutanoyl)isoswertisin.

**Fig 8:**
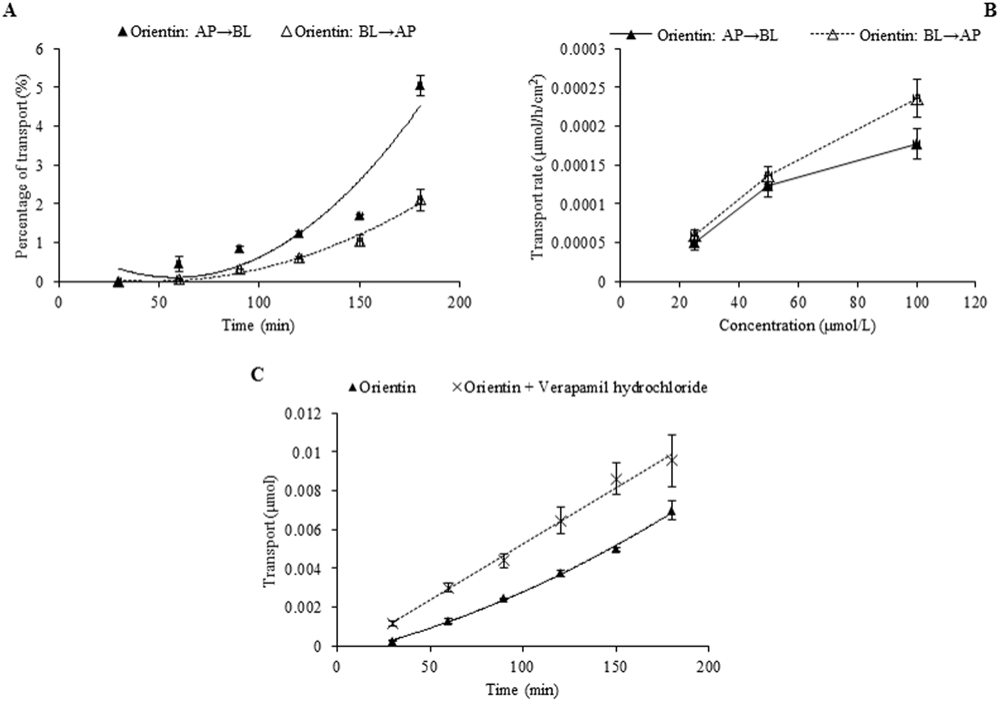
Transport of orientin across Caco-2 cell monolayer. Notes: (A) The percent transport of orientin as a function of time at 50 μmol/L; (B) The transport rate of orientin as a function of concentration at 90 min; (C) The effect of verapamil hydrochloride on the transport of orientin as a function of time at 50 μmol/L.

**Fig 9:**
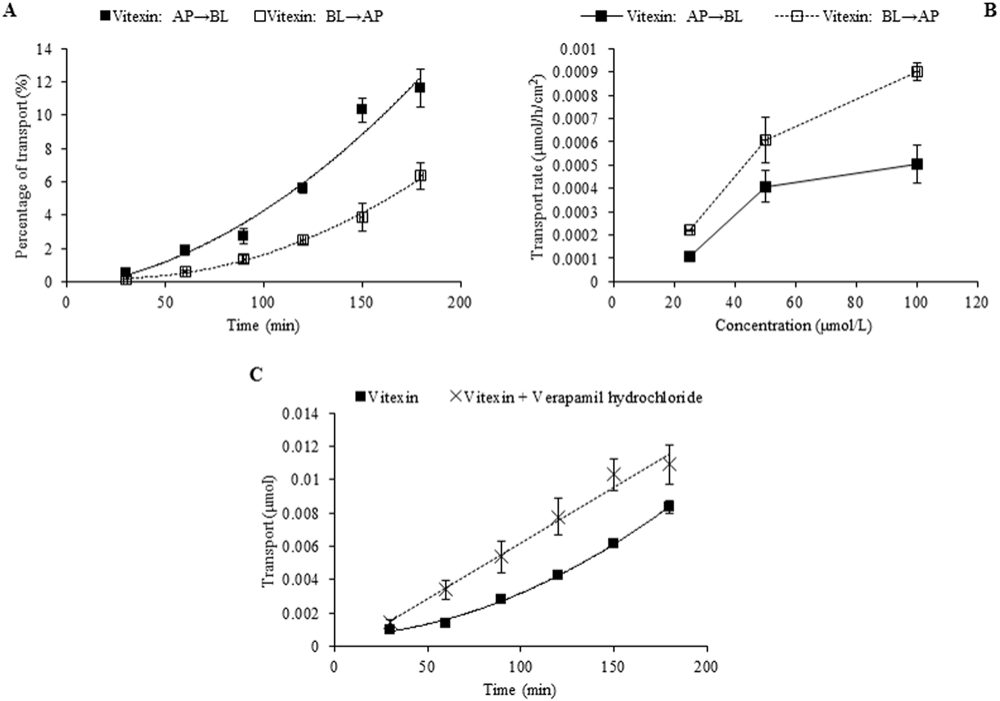
Transport of vitexin across Caco-2 cell monolayer. Notes: (A) The percent transport of vitexin as a function of time at 50 μmol/L; (B) The transport rate of vitexin as a function of concentration at 90 min; (C) The effect of verapamil hydrochloride on the transport of vitexin as a function of time at 50 μmol/L.

**Fig 10:**
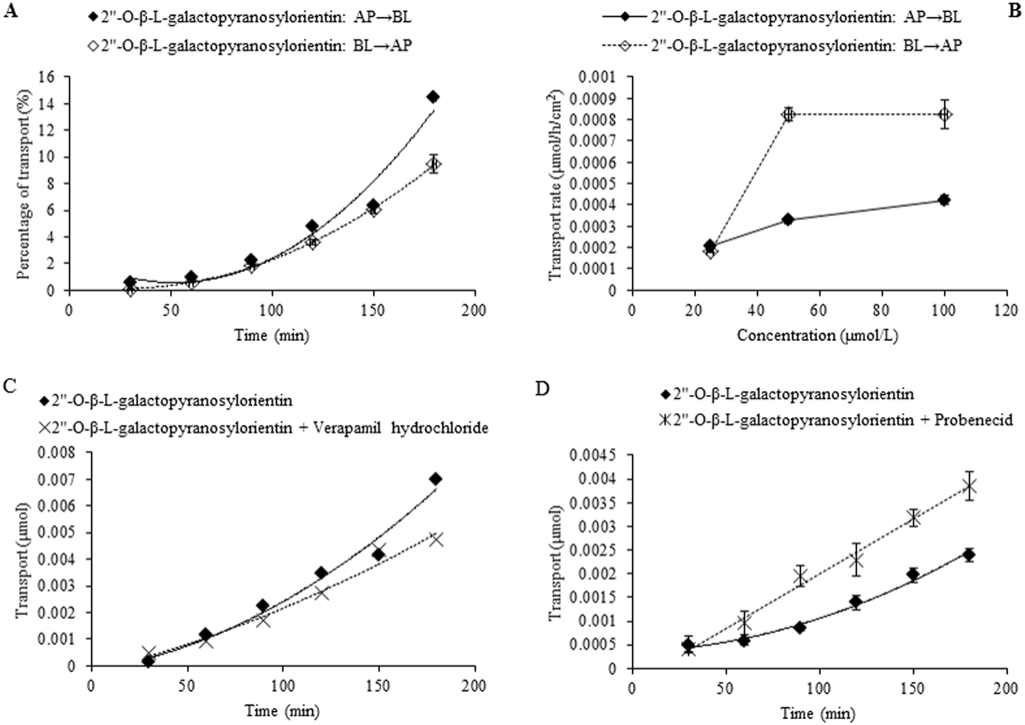
Transport of 2”-*O*-β-L-galactopyranosylorientin across Caco-2 cell monolayer. Notes: (A) The percent transport of 2”-*O*-β-L-galactopyranosylorientin as a function of time at 50 μmol/L; (B) The transport rate of 2”-*O*-β-L-galactopyranosylorientin as a function of concentration at 90 min; (C) The effect of verapamil hydrochloride on the transport of 2”-*O*-β-L-galactopyranosylorientin as a function of time at 50 μmol/L; (D) The effect of probenecid on the transport of 2”-*O*-β-L-galactopyranosylorientin as a function of time at 50 μmol/L.

**Fig 11:**
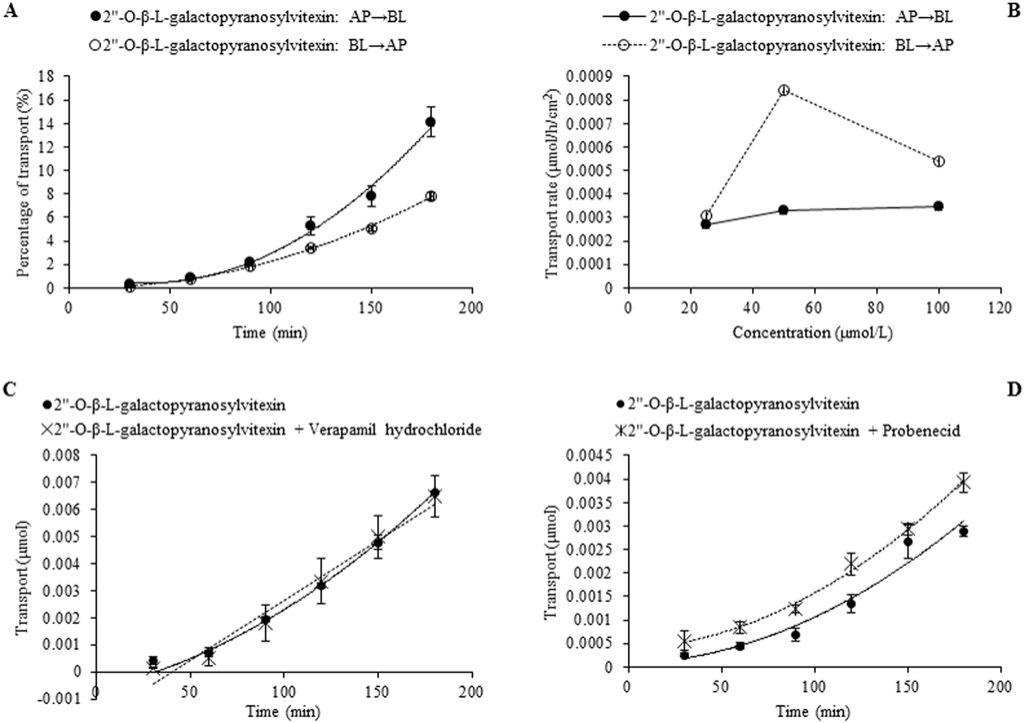
Transport of 2”-*O*-β-L-galactopyranosylvitexin across Caco-2 cell monolayer. Notes: (A) The percent transport of 2”-*O*-β-L-galactopyranosylvitexin as a function of time at 50 μmol/L; (B) The transport rate of 2”-*O*-β-L-galactopyranosylvitexin as a function of concentration at 90 min; (C) The effect of verapamil hydrochloride on the transport of 2”-*O*-β-L-galactopyranosylvitexin as a function of time at 50 μmol/L; (D) The effect of probenecid on the transport of 2”-*O*-β-L-galactopyranosylvitexin as a function of time at 50 μmol/L.

In the case of individual transport experiments, the ratios of P_app AP→BL_ to P_app BL→AP_ of isoswertisin, isoswertiajaponin, and 2”-*O*-(2”‘-methylbutanoyl)isoswertisin were almost equal to 1, while those of the remaining four test compounds were below 0.5 (Table 2). However, the same ratios of these compounds except 2”-*O*-(2”‘-methylbutanoyl)isoswertisin were increased when tested in the equimolar mixture (Table 3). No matter tested individually or in the equimolar mixture, the bidirectional transport percentages of isoswertisin, isoswertiajaponin, and 2”-*O*-(2”‘-methylbutanoyl)isoswertisin increased approximately linearly with time (Figs. 3–6), and their transport rates also increased with concentration in the range of 25–100 μmol/L in an approximately linear manner at 90 min (Figs. 3–5, 7). In contrast, the time course and concentration course for trans-monolayer transport of orientin, vitexin, 2”-*O*-β-L-galactopyranosylorientin, and 2”-*O*-β-L-galactopyranosylvitexin showed that their bidirectional transport percentages and transport rates were increased in a non-linear manner with the time and concentration in both individual state and the equimolar mixture (Figs. 6–11). Nevertheless, the bidirectional transport percentages of these compounds except 2”-*O*-(2”‘-methylbutanoyl)isoswertisin were decreased in the equimolar mixture compared with in individual state, and those from BL to AP direction were decreased dramatically in the equimolar mixture (Fig. 6). As for 2”-*O*-(2”‘-methylbutanoyl)isoswertisin, although the transport percentage of AP to BL direction in the equimolar mixture was decreased as much as that of the other six compounds, the value of BL to AP direction in the equimolar mixture was increased almost twice compared with that in individual state (Fig. 6).

### Transporter inhibition experiment

The results of time course and concentration course of trans-monolayer transport and the influences of verapamil hydrochloride and probenecid on the transport of orientin, vitexin, 2”-*O*-β-L-galactopyranosylorientin, and 2”-*O*-β-L-galactopyranosylvitexin are exhibited in Figs. 8–11. As for orientin and vitexin, the bidirectional transports and transport rates were increased in non-linear manner with the time and concentration when they were transported alone, while their quantities transported from AP to BL sides increased dramatically (*P*<0.05) and almost in linear manner with incubation time when they were transported along with verapamil hydrochloride, respectively (Figs. 8 and 9). As for 2”-*O*-β-L-galactopyranosylorientin and 2”-*O*-β-L-galactopyranosylvitexin, their non-linear transport was almost unchanged in the presence and absence of verapamil hydrochloride, but the transport of the former other than the latter was changed to linear after co-transported with probenecid (Figs. 10 and 11).

### LogP values

The calculated logP values of the seven flavonoids are presented in Table 2.

## Discussion

The well-recognized Caco-2 cell monolayer model was employed to investigate the intestinal absorption of the seven flavonoids from the flowers of *T*. *chinensis* in this study. Based on the bidirectional P_app_ values, six out of the seven flavonoids were assessed as hard absorbable compounds because their values were at the same level as that of the hard absorbable standard compound atenolol [20–23]. The remaining compound, 2”-*O*-(2”‘-methylbutanoyl)isoswertisin could be assessed as moderate absorbable compound because it gave a P_app_ value (at the level of 10^-6^) between those of atenolol and propranolol. The latter is usually considered as the easily absorbable standard compound [21–23]. This was also supported by their LogP values (Table 2), because it is generally considered that the compound with LogP in the range 1–3 is optimum for absorption [24]. In this case, all test compounds, except 2”-*O*-(2”‘-methylbutanoyl)isoswertisin which has a LogP value of 1.17, have negative LogP values. Undoubtedly, the good absorbability of 2”-*O*-(2”‘-methylbutanoyl)isoswertisin is ascribed to the lipophilic methylbutyryl substituent at R_3_ (Fig. 1), which reduces the polarity of the whole molecule.

The seven flavonoids can be categorized into two large groups on the basis of their absorption mechanism. One group is composed of isoswertisin, isoswertiajaponin, and 2”-*O*-(2”‘-methylbutanoyl)isoswertisin. Their almost equal bidirectional transport demonstrated by the ratios of P_app AP→BL_ to P_app BL→AP_ (close to 1) (Table 2) and the linear transport percentage over time and concentration demonstrated by time course and concentration course (Figs. 3–5) indicated that they were absorbed mainly through passive diffusion without participation of transporter. Their absorbability in descending order was 2”-*O*-(2”‘-methylbutanoyl)isoswertisin > isoswertisin > isoswertiajaponin on the basis of their P_app_ values, which was consistent with their LogP values in the same order (Table 2). Therefore, the lipo-hydro partition coefficient or molecular polarity was the key determinant for the absorption of these three flavonoids. The remaining four compounds constituted another group of which the absorption involved transporter mediated active efflux in addition to passive diffusion, which was evidenced by their ratios of P_app AP→BL_ to P_app BL→AP_ being less than 0.8 even below 0.5 (Table 2) and non-linear transport with time and concentration demonstrated by the time course and concentration course (Figs. 8–11). The smaller ratios of P_app AP→BL_ to P_app BL→AP_ showed that the transport from AP to BL sides was slower than that from BL to AP sides due to the participation of efflux transporter(s) at AP side, which is corresponding to the lumen side of intestine. Their non-linear time course of transport showed an uptrend with incubation time (Figs. 8–11), which may be explained by the saturation of efflux transporter(s) resulting in the relative increase of transport. To further confirm the presence of efflux transporter, the inhibition experiment was carried out. As well known, P-gp and MRP [25] are important efflux transporters which decrease the quantities transported and bioavailability of drugs, and verapamil hydrochloride and probenecid can inhibit P-gp and MRP2 mediated efflux, respectively [26, 27]. The results demonstrated that verapamil hydrochloride increased the transports of orientin and vitexin and made their transport curve over time into a line (Figs. 8 and 9), but had no effect on the transports of 2”-*O*-β-L-galactopyranosylorientin and 2”-*O*-β-L-galactopyranosylvitexin (Figs. 10 and 11). Thus, it can be concluded that both orientin and vitexin rather than 2”-*O*-β-L-galactopyranosylorientin and 2”-*O*-β-L-galactopyranosylvitexin are the substrates of P-gp. As for probenecid, it significantly promoted the transport of 2”-*O*-β-L-galactopyranosylorientin but had no influence on that of 2”-*O*-β-L-galactopyranosylvitexin (Figs. 10 and 11). Therefore, 2”-*O*-β-L-galactopyranosylorientin rather than 2”-*O*-β-L-galactopyranosylvitexin can be considered as the substrate of MRP2.

Based on the structure of the seven compounds, it is found that each of the three flavonoids absorbed through passive diffusion, i.e., isoswertisin, isoswertiajaponin, and 2”-*O*-(2”‘-methylbutanoyl)isoswertisin has a methoxyl group at C-7 of the skeleton (Fig. 1, R_2_ = CH_3_), while each of the remaining four compounds of which the absorption involves active transport has a hydroxyl group at C-7 (Fig. 1, R_2_ = H). Thus, the existence of the hydroxy or methoxy at C-7 is the key factor for the transport with or without efflux. This induction has nothing with taking a part for the whole because the only difference between the compounds with active efflux and the compounds without active efflux, such as between vitexin and isoswertisin, as well as between orientin and isoswertiajaponin, is the hydroxy or methoxy at C-7. It is also found that the substrates of P-gp, i.e., orientin and vitexin both have a free hydroxyl group at C-2” of the flavone skeleton (Fig. 1, R_3_ = H), while the nonsubstrates of P-gp, i.e., 2”-*O*-β-L-galactopyranosylorientin and 2”-*O*-β-L-galactopyranosylvitexin, have a *O*-galactopyranosyl group at this position (Fig. 1, R_3_ = β-L-galactopyranosyl). Therefrom, the unbound state of 2”-OH determines the involvement of the efflux mediated by P-gp. In addition, the hydroxyl group at C-2’ of the flavone skeleton may influence the involvement of MRP2 in the transport because 2”-*O*-β-L-galactopyranosylorientin (Fig. 1, R_1_ = OH) instead of 2”-*O*-β-L-galactopyranosylvitexin (Fig. 1, R_1_ = H) is the substrate of MRP2. In summary, the following rules in priority order about the structure-absorbability relationship of these seven flavonoids could be generalized. At first, the hydroxyl or methoxyl group at C-7 determines the involvement of efflux transporter(s); secondly, the free state of hydroxyl group at C-2” determines the involvement of P-gp; and finally, the hydroxyl group at C-2’ determines the involvement of MRP2.

In order to investigate the interaction of the compounds during absorption, the transport experiment of the seven flavonoids in an equimolar mixture was carried out. When they were mixed together, the percentages of permeation of these compounds except 2”-*O*-(2”‘-methylbutanoyl)isoswertisin were decreased, especially from BL to AP sides, and the reductions had a positive correlation with their polarities. Thus, it can be assumed that the linearity of passive diffusion is only limited to a concentration range, and the combined concentration of the mixed compounds exceeds this linear range. For this reason, the main decrease of the six out of the seven compounds was from BL to AP sides, resulting in the increase of the ratios of P_app AP→BL_ to P_app BL→AP_. As for 2”-*O*-(2”‘-methylbutanoyl)isoswertisin, which is the least polar compound, the ratio was low to 0.25 due to the increased permeation from BL to AP sides and the reduction from AP to BL sides. This might be ascribed to its least polarity, which enabled it to have an advantage in the competition for passive diffusion, and the shortage of transporter in BL side. Interestingly, although the permeation of 2”-*O*-(2”‘-methylbutanoyl)isoswertisin was dominated in the transport experiments of individual compounds, it was not the case in the transport experiment of the mixture, indicating that the compound interaction makes a difference to the absorption of individual compounds.

## Conclusions

In conclusion, the representative seven flavonoids from the flowers of *T*. *chinensis* are hard to be absorbed either in individual state or in the mixture, and their absorption mechanism usually involves transporter mediated active efflux in addition to passive diffusion depending on the specific substituents at C-7, C-2”, and C-2’. The results of this study also imply that the absorbability of the flavonoids should be taken into account when estimating the effective components of *T*. *chinensis*.
